# Effects of Hydroxytyrosol Supplementation on Performance, Fat and Blood Parameters of Broiler Chickens

**DOI:** 10.3390/ani14010119

**Published:** 2023-12-28

**Authors:** Kelly M. M. Dias, Carlos H. Oliveira, Arele A. Calderano, Horacio S. Rostagno, Kevin E. O’Connor, Reeta Davis, Meg Walsh, James Britton, Enrico A. Altieri, Luiz F. T. Albino

**Affiliations:** 1 Department of Animal Science, Federal University of Viçosa, Viçosa 36570-900, Minas Gerais, Brazil; carlos.oliveira2@ufv.br (C.H.O.); calderano@ufv.br (A.A.C.); rostagno@ufv.br (H.S.R.); lalbino@ufv.br (L.F.T.A.); 2 Nova Mentis Limited, Nova UCD, Belfield Innovation Park, University College Dublin, Belfield, D04 F438 Dublin, Ireland; koconnor@novamentis.eu (K.E.O.); rdavis@novamentis.eu (R.D.); mwalsh@novamentis.eu (M.W.); jbritton@novamentis.eu (J.B.); ealtieri@novamentis.eu (E.A.A.)

**Keywords:** antioxidant, growth performance, lipid composition, liver damage, feed additives

## Abstract

**Simple Summary:**

Modern broilers are vulnerable to various stressor agents which may disturb their antioxidant system and lead to poor performance. Supplementation with feed additives which can improve broilers’ antioxidant response can be an effective nutritional intervention. This study evaluated the performance, fat, and blood parameters of broilers fed diets supplemented with the antioxidant hydroxytyrosol. Hydroxytyrosol supplementation improved meat quality through the reduction of fatty acid content of breast meat and, moreover, improved liver function through the reduction of enzymes related to liver health. Therefore, hydroxytyrosol supplementation can be used as a potent feed additive capable of reducing the oxidative stress caused by the intensive production system.

**Abstract:**

The study aimed to evaluate the effects of dietary supplementation of hydroxytyrosol (HT) on performance, fat, and blood parameters of broilers. In total, 960 male chicks were distributed into four treatments groups with 12 replicates with 20 birds per pen, with varying HT levels (0, 5, 10, and 50 mg/kg of feed) added to the basal diet from 1 to 42 days old. Feed intake, body weight gain, and feed conversion ratio were evaluated. Enzymes related to liver injury were evaluated in blood. Fatty acid profile and malondialdehyde (MDA) concentration were determined in the breast meat. Dietary supplementation of HT did not improve broilers’ performance (*p* > 0.05). Birds fed 50 mg HT/kg had lower AST, ALT, and GGT concentrations (*p* ≤ 0.05), whereas broilers fed 5, 10, and 50 mg HT/kg, had lower TBIL concentrations (*p* ≤ 0.05). Breast meat of broilers fed 50 mg HT/kg had lower lipid content, saturated fatty acid, unsaturated fatty acids, MDA concentrations (*p* ≤ 0.05), and polyunsaturated fatty acids (*p* < 0.0001). In summary, supplementation of 5, 10, and 50 mg HT/kg does not improve the performance of broilers, but the dose of 50 mg HT/kg helps the liver against inflammation and improves fat parameters.

## 1. Introduction

The intensive feeding and the pursuit of rapid growth in modern broilers make them vulnerable to oxidative stress resulting from high temperatures, diseases, and exposure to toxic substances from water and feed [1,2]. The oxidative stress can lead to poor performance, compromise health status, and reduce the meat quality of broiler chickens [3,4]. However, nutritional interventions, such as the use of antioxidants, represent an effective method to improve oxidative stress responses [5,6].

Polyphenols are well-considered and consumed by the general population due to their capacity to prevent and treat different diseases [7]. Hydroxytyrosol (HT) is a natural polyphenol found in olives that has strong antioxidant activity. It also can improve endothelial dysfunction, the lipid profile of adipocytes and blood metabolites, and anti-inflammatory properties have been observed in humans [8,9].

HT primarily functions as a radical scavenger, and its role as an immunity enhancer has been positively reported in broilers. Moreover, it is associated with the downregulation of genes related to sterol metabolism and fat deposition, the reduction of blood lipid levels, an increase in the antioxidant capacity of broiler meat, and enrichment of meat in monounsaturated fatty acids [10,11,12,13]. In addition, its effectiveness in reducing *Campylobacter* spp. in broiler excreta has been reported [10], which is an important factor contributing to foodborne diseases. However, these effects have been evaluated mainly through the use of olive byproducts, such as extra-virgin olive oil, dried olive pulp, olive oil mill wastewater, and destoned “paté” olive cake, which suffer from batch-to-batch and seasonal variation. HT can be extracted from olive fruit, oil, and leaves, or it can also be synthetically produced, being considered a stable compound in its free form [14,15]. Thus, it may be a promising antioxidant in poultry nutrition.

The hypothesis of this study was that broilers fed with HT would exhibit enhanced performance parameters, improved blood health indicators, and positive alterations in fat parameters. Therefore, the objective of the present study was to evaluate the effects of dietary HT supplementation on performance, investigate the influence on fat aiming to understand its effect on lipid metabolism, and assess blood parameters to elucidate potential physiological changes of broiler chickens from 1 to 42 days old. 

## 2. Materials and Methods

### 2.1. Birds, Experimental Design, and Diets 

The experiment was carried out at the Teaching, Research and Extension Unit in Poultry Nutrition and Production of the Department of Animal Science, Federal University of Viçosa, Viçosa, Minas Gerais, Brazil.

Cobb500™ male broiler chicks were obtained from a commercial hatchery (Rivelli Alimentos SA, Matheus Leme, MG, Brazil), where all chicks received vaccinations for Marek and Newcastle diseases, and infectious bronchitis.

A total of 960 one-day-old Cobb500™ male chicks (48.0 g ± 2.69 g) were weighed and randomly distributed into four treatments groups with 12 replicates with 20 birds per pen (35 kg/m^2^). Birds were housed in floor pens (1 × 2 m) equipped with five in-line nipple drinkers, a tubular feeder, and covered with wooden shavings. Free access to feed and water was guaranteed during the experimental period. The temperature was initially set at 33 °C and gradually decreased until reached 20 °C. The lighting program was set to 24 h for the first week, then reduced to 16 h of light from 7 to 42 days.

The 42-day trial was divided into two phases: (1) starter, 1 to 21 days, and (2) grower/finisher, 21 to 42 days old. The treatments consisted of a basal diet supplemented with four levels of HT (0, 5, 10, and 50 mg/kg of feed). After conducting a literature review, HT levels were determined. HT was mainly used through olive byproducts. The total amount of HT found in those coproducts was calculated to determine the doses used in the present study. The dose of 10 mg HT/kg of feed was the most common. Lower and higher doses were determined to evaluate the effectiveness and to test the toxicity of HT. The source of HT used was 1-HT^®^ hydroxytyrosol (>98% pure; Nova Mentis Ltd., Dublin, Ireland). 

The basal diet was based on corn and soybean meal and formulated to meet the requirements of the birds according to Rostagno et al. [16] (Table 1). To form the experimental diets, first, the basal diet was completely mixed. Subsequently, approximately 2 kg of this basal diet was premixed with HT, and then, this mixture was incorporated into the mixer containing the remaining basal diet and were homogeneously mixed. This process was repeated for each treatment containing HT in each rearing phase.

### 2.2. Performance Parameters

Birds and feed were weighed at the beginning and end of the experimental phases to determine feed intake (FI, kg/bird), body weight (BW, kg/bird), BW gain (BWG, kg/bird), and feed conversion ratio (FCR, kg/kg). In case of mortality, the leftover feed was weighed to correct FI.

### 2.3. Samples Collection

At the end of the trial (42 days), all birds were weighed and then two birds with the closest BW to the average weight of each replicate (totaling 24 birds per treatment) were selected for sample collection. One of the birds was destined only for blood samples, but both breasts were collected for further analysis. The selected birds had a fasting period of 12 h.

#### 2.3.1. Blood Metabolites

On the 42nd day, after the fasting period, blood samples were collected from one of the selected birds, placed in CAT Serum Activator Coagulation vacuum tubes, and immediately sent for analysis. The analyses were alanine aminotransferase (ALT; U.V. kinetic method), aspartate aminotransferase (AST; U.V. kinetic method), alkaline phosphatase (ALP; kinetic method—IFCC), gamma-glutamyl transpeptidase (GGT; kinetic method—Szasz modified), total bilirubin (TBIL; dichloroaniline method), cholesterol (enzymatic colorimetric method), high-density lipoprotein (HDL; oxidase cholesterol—direct method), low-density lipoprotein (LDL; enzymatic colorimetric method), very low-density lipoprotein (VLDL; enzymatic colorimetric method), and triglyceride (enzymatic colorimetric method) which were performed by ViçosaLab (Viçosa, Minas Gerais, Brazil).

#### 2.3.2. Breast Meat Composition

On the 42nd day, after the fasting period, birds were slaughtered by cervical dislocation, and breast samples were removed for further analysis. Breast meat samples were analyzed for lipid content (Methods 14 and 16 [17]), fatty acids profile (gas chromatography—PA 006), and peroxide index (Method 33 [17]) by Mérieux NutriSciences, São Paulo, São Paulo, Brazil, and for malondialdehyde (MDA; Modified Aqueous Extraction Method MA-114 R.0 [18]) by CBO Análises Laboratoriais, Valinhos, São Paulo, Brazil. Some details regarding the analysis can be found below.

##### Lipid Content

Samples were treated under heating with hydrochloric acid. The mixture is cooled and filtered. After washing and drying, the remaining sample is submitted for extraction with light petroleum. Samples were weighed (2.5 g ± 0.5 g), placed in an Erlenmeyer flask, 50 mL of hydrochloric acid 40% was added, and the mixture was agitated. The Erlenmeyer flask was fit in the reflux condenser, and after the mixture started boiling the water reflux was set for 45 min. The mixture is cooled and filtered through a moistened, fat-free, double filter paper. The double filter paper was placed on a watch glass and dried for an hour in the air oven at 100 °C ± 3 °C. Then, the double filter containing the dry residue was evolved into another double filter to avoid losses. Finally, the extraction was performed using the Soxhlet method.

##### Fatty Acid Profile

Samples were converted to a fatty acid methyl ester (FAME) derivative. FAMEs from samples were extracted using hexane solvent, placed into a non-screw-cap disposable glass tube, and dried under nitrogen. Then, the free cholesterol was removed to avoid contamination of the samples. Dry hexane was added to the FAMEs which were transferred into the GC auto sample vial. FAMEs were analyzed using gas chromatography.

##### Peroxide Index

The method applies to samples with a peroxide index of up to 70 meq/kg. Briefly, 2–5 g of samples were weighed, and placed in an Erlenmeyer flask (250 mL), where 50 mL of acetic acid: isooctane 3:2 solution was added. The mixture was agitated and had 0.5 mL of potassium iodate saturated solution added to it. The mixture rested for a minute, away from the light, and 30 mL of water was added at the end. Then, a titration of the mixture with 0.1 mol/L of sodium thiosulfate solution was made until the yellowish coloring disappeared. In addition, 0.5 mL of SDS 10% was added to the titration, and after, 1 mL of starch indicator solution. Finally, sodium thiosulfate solution (0.1 mol/L drop) was added until the bluish color disappeared.

### 2.4. Statistical Analysis

Data were analyzed by one-way ANOVA using ExpDes.pt from the R statistical package (R Software v.4.0.4) and means were compared using a Tukey test. Differences were considered significant at α = 0.05. Each replicate was considered an experimental unit. 

## 3. Results

### 3.1. Performance

In the starter phase (1 to 21 days), only FI was influenced by HT supplementation, in which, birds fed 5 mg/kg of HT had a higher FI than birds fed 10 mg/kg of HT (*p* = 0.03; Table 2). There was no influence of HT levels on BWG, BW, and FCR in the grower/finisher phase (21 to 42 days) and in the overall period (1 to 42 days; *p* > 0.05).

### 3.2. Blood Metabolites

Regardless of the level, birds fed HT had lower TBIL concentrations when compared to birds that were not fed HT (*p* < 0.001; Table 3). Birds fed 50 mg/kg of HT had lower levels of AST (*p* = 0.003), ALT (*p* < 0.0001), and GGT (*p* < 0.0001). However, no effects of HT on ALP, cholesterol, HDL, LDL, VLDL, and triglyceride concentrations were observed (*p* > 0.05).

### 3.3. Breast Meat Composition

Breast meat of birds fed 50 mg/kg of HT had lower concentrations of lipids (*p* = 0.022), palmitic acid (*p* = 0.015), stearic acid (*p* = 0.025), oleic acid (*p* = 0.004), saturated fatty acid (SFA; *p* = 0.024), omega 9 fatty acids (ω-9; *p* = 0.004), total fatty acids (*p* = 0.009), unsaturated fatty acids (*p* = 0.004), and MDA (*p* = 0.018) when compared to birds that were not fed HT. Regardless of the level, HT inclusion reduced the concentrations of linoleic acid (*p* < 0.001), polyunsaturated fatty acids (PUFA; *p* < 0.0001), and omega 6 fatty acids (ω-6; *p* < 0.0001) in breast meat. Moreover, birds fed 10 or 50 mg/kg of HT had lower concentrations of monounsaturated fatty acids (MUFA; *p* = 0.03) and cis-MUFA (*p* = 0.003) when compared to birds that were not fed HT (Table 4). However, HT had no effect on the peroxide index (*p* = 0.323). Other FA evaluated and not mentioned had their values lower than the quantification limit (Table S1).

## 4. Discussion

The main polyphenol component found in olive and in its different byproducts is HT. The effects of HT on broiler chickens have been described through the use of olive products such as extra-virgin olive oil, dried olive pulp, olive oil mill wastewater, and destoned “paté” olive cake [10,12,19,20,21]. However, HT in its isolated and pure form has not been tested. For example, dried olive pulp is described to have around 220 mg of HT per kg, whereas extra-virgin olive oil contains around 42 mg of HT per kg; however, the use of by-products shows inconsistent results mainly due to the variation in composition, especially regarding the fiber content [12,20,21]. In the current research, dietary supplementation of HT in free form did not affect growth performance of broiler chickens. The reason for this may be related to the fact that birds were reared under low level of stress and good sanitary conditions. It has been reported that phenolic compounds are more likely to present beneficial effects on performance and immune response when animals are subjected to stressful conditions [11,13,21,22]. In line with our findings, utilization of antioxidant in broiler diets, such as selenium and vitamin E, had no effect on the performance of broilers reared under normal conditions [23,24]. On the other hand, increasing dried olive pulp levels in broiler diets resulted in a worse FCR, which was explained mainly by the high fiber content of this by-product [12,21]. However, adding extra-virgin olive oil as an oil source promoted better BW and FCR compared to lard and sunflower oil [20], and increasing destoned “paté” olive cake levels improved BW and FCR compared to a control diet without it [10]. These results were related to olive oil polyphenols action on the digestibility caused by the reduced digesta passage rate.

Blood analysis of some metabolites may help in assessing broiler health status. Serum concentrations of ALT, AST, GGT, and TBIL are commonly used as indicators of liver damage and function. ALT is an enzyme that contributes to cellular nitrogen metabolism and gluconeogenesis by catalyzing the conversion of alanine and α-ketoglutarate to pyruvate and glutamate, whereas AST is an enzyme that is responsible for catalyzing the reversible transfer of amino acids groups between glutamate and aspartate [25]. It is well-established that higher serum concentrations of AST and ALT are correlated with liver inflammation and fat accumulation [26,27,28]. Therefore, a reduction in serum concentration of both enzymes means that there are improvements in protection against oxidative damage by improving redox status [26]. In line with that, GGT can be correlated to degenerative changes in the liver, and it also plays a major role in glutathione degradation, contributing to the maintenance of cellular glutathione levels. This in turn enhances cellular resistance to hydrogen peroxide injury [29,30]. Thus, lower GGT serum concentration suggests that the antioxidant capacity is increased, since there is less glutathione degradation. Effects of HT supplementation showed that broiler chickens fed 50 mg/kg had lower serum concentrations of AST, ALT, and GGT between the treatments tested, showing that HT has an important role in the protection against liver oxidative damage and inflammation. Contrary to our findings, some studies reported that the utilization of olive by-products did not alter serum concentrations of AST, ALT, and GGT [21,31], which can be attributed to the difficulty in achieving high levels of HT through olive by-products. In line with this idea, supplementation with Se reduced AST and ALT serum concentrations in broilers [24,26], indicating the role of antioxidants in liver protection. Moreover, a high presence of TBIL in blood indicates liver injury and hemoglobin degradation, and it includes direct bilirubin and indirect bilirubin [32,33]. In the present study, birds fed HT, regardless of the level, showed lower serum concentrations of TBIL compared to the group without HT supplementation; thus, strengthening the evidence that HT acted to mitigate liver injury.

The FA composition and antioxidant capacity are some of the important determinants of meat quality [31,34]. The main fatty acids in chicken meat are C16:0, C18:0, C18:1n9c, and C18:2n6c [35]. High dietary SFA intake, especially myristic acid (C14:0) and palmitic acid (C16:0), is positively correlated to coronary artery disease due to its hypercholesterolemic effect [34,36,37]. On the other hand, PUFA content in meat is desired by consumers; however, they are one of the reasons for quality degradation during storage due to their great susceptibility to oxidation processes and lipid oxidation in meat [38,39]. The fatty acid profile in broiler meat was found to have a correlation with the feed composition, suggesting that it is possible to manipulate these parameters using different ingredients and/or additives [39]. In the present study, a reduction in SFA in breast meat of the group fed 50 mg HT/kg of feed, compared to the group without HT, was observed, which should be attributed to the reduction in C16:0 and C18:0. In addition, HT supplementation regardless of the level reduced C18:2n6c, which has been reported to cause a negative flavor in beef and chicken meat [40,41]. In the same way, HT supplementation decreased PUFA content. Sabino et al. [11] performed a nutrigenomic evaluation of supplementary olive oil mill wastewater on the broiler’s jejunum. Among the functional genes identified, the authors reported that olive oil mill wastewater had a direct effect on lipid metabolism through the downregulation of genes such as farnesyl diphosphate synthase, NAD(P) dependent steroid dehydrogenase-like, and farnesyl-diphosphate farnesyltransferase 1, which are involved in diverse processes of fat deposition in chickens, therefore suggesting that olive oil mill wastewater could have a beneficial effect in reducing fatty acid transportation with a decrease in body fat accumulation. Since the most abundant polyphenol compound found in olive oil mill wastewater is HT, these beneficial effects could be associated with its function [42,43], which might explain the reduction in both saturated and unsaturated fatty acids, lipid content, and the results of liver enzymes that are related to fat accumulation. In summary, HT supplementation had a positive influence on breast meat quality for human consumption and might benefit meat flavor. Papadomichelakis et al. [12] reported that offering a combination of 50 and 80 g dried olive pulp/kg, in the grower and finisher phases, respectively, reduced SFA content, and increased MUFA in broilers’ breast meat. Moreover, Mohebodini et al. [34] supplementing Eucalyptus globulus essential oil in broiler diets observed that SFA, C16:0, and C18:0 linearly decreased, whereas PUFA and C18:2 linearly increased in thigh muscle.

The primary cause of decreased meat quality is lipid oxidation caused by high levels of free radicals. Lipid peroxidation induces oxidative stress, increases MDA content, and causes meat deterioration [23,31]. MDA is a stable end-product of lipid peroxidation and is widely used as an indicator of cumulative lipid peroxidation [44]. In the present research, breast meat of birds fed 50 mg HT/kg of feed had lower MDA concentrations than the group without HT, which suggests that HT might act by reducing lipid peroxidation and therefore, improving meat quality. Similar to our findings, the highest doses of HT through the utilization of olive by-products, such as destoned “paté” olive cake, olive oil mill wastewater, extra-virgin olive oil, and dried olive pomace, lead to a reduction in MDA concentrations in broiler meat and blood [10,19,20,31]. The authors interpreted their results as a consequence of the presence of phenolic compounds, such as HT, in the by-products utilized. HT has high antioxidant activity and can inhibit meat oxidation through the donation of H+ to alkyl peroxyl radicals generated by lipid oxidation, therefore resulting in better oxidative stability [10,31].

## 5. Conclusions

Dietary supplementation of 5, 10, and 50 mg HT/kg of feed does not improve the performance of broilers. However, the dose of 50 mg HT/kg helps the liver against damage and inflammation and also acts on meat quality through the improvement of fatty acid profile and reduction of lipid oxidation. Therefore, the dose of 50 mg HT/kg of feed is recommended to improve broilers’ health. However, we believe that further studies should be carried out to better understand the mechanisms in which HT plays its role in broiler metabolism.

## Figures and Tables

**Table 1:** Ingredients and calculated nutritional composition of the basal diets (as-fed basis).

Ingredient, %	Basal Diet (1 to 21 Days)	Basal Diet (21 to 42 Days)
Corn, 7.88%	52.06	57.48
Soybean meal, 45.0%	41.18	35.05
Soybean oil	3.05	4.23
Dicalcium phosphate	1.24	0.93
Limestone	1.01	0.79
Salt	0.52	0.49
DL-Methionine, 99%	0.317	0.272
L-Lysine HCl, 78%	0.140	0.283
L-Threonine, 98.5%	0.047	0.037
Choline chloride, 60%	0.100	0.100
Phytase (100 g/ton), 500 FTU/kg	0.010	0.010
BHT ^3^	0.010	0.010
Mineral Premix ^1^	0.130	0.130
Vitamin Premix ^2^	0.130	0.130
Calculated nutritional composition ^4^		
Crude Protein, %	23.24	20.89
Metabolizable Energy, kcal/kg	3000	3150
Calcium, %	0.937	0.758
Available Phosphorus, %	0.440	0.374
Sodium, %	0.218	0.208
SID Lysine, %	1.256	1.124
SID Methionine, %	0.624	0.554
SID Methionine + Cysteine, %	0.929	0.832
SID Threonine, %	0.829	0.742
SID Tryptophan, %	0.267	0.235

^1^ Trace mineral premix provided per kg of diet: Mn, 58.36 g; Fe, 41.68 g; Zn, 54.21 g; Cu, 8.31 g; I, 0.84 g; Se, 0.25 g. ^2^ Vitamin premix provided per kg of diet: vitamin A, 9,638,000 IU; vitamin D3, 2,410,000 IU; vitamin E, 36,100 IU; vitamin B1, 2.60 g; vitamin B2, 6.45 g; vitamin B6, 3.61 g; vitamin B12, 15.9 mg; vitamin K3, 1.94 g; pantothenic acid, 12.95 g; nicotinic acid, 39.20 g; folic acid, 0.90 g; biotin, 89.80 mg. ^3^ Antioxidant Butylhydroxytoluene. ^4^ Values calculated according to Rostagno et al. [16].

**Table 2:** Feed intake (FI), body weight gain (BWG), body weight (BW), and feed conversion ratio (FCR) of broiler chickens fed different levels of hydroxytyrosol.

	0 mg HT/kg	5 mg HT/kg	10 mg HT/kg	50 mg HT/kg	*p*-Value	SEM
Starter phase—1 to 21 days						
FI, kg/bird	1.449 ^ab^	1.471 ^a^	1.437 ^b^	1.463 ^ab^	0.032	0.004
BWG, kg/bird	1.145	1.155	1.143	1.154	0.384	0.003
BW, kg/bird	1.192	1.201	1.191	1.201	0.465	0.003
FCR, kg/kg	1.265	1.274	1.257	1.267	0.410	0.003
Grower/Finisher phase—21 to 42 days						
FI, kg/bird	3.909	3.956	3.947	3.932	0.733	0.016
BWG, kg/bird	2.408	2.450	2.449	2.438	0.200	0.008
BW, kg/bird	3.601	3.651	3.639	3.631	0.263	0.010
FCR, kg/kg	1.624	1.615	1.612	1.613	0.902	0.006
Overall period—1 to 42 days						
FI, kg/bird	5.357	5.427	5.384	5.395	0.555	0.017
BWG, kg/bird	3.553	3.604	3.592	3.593	0.222	0.009
BW, kg/bird	3.601	3.651	3.646	3.639	0.179	0.009
FCR, kg/kg	1.508	1.506	1.493	1.502	0.563	0.004

^a,b^ Means followed by different letters in the same row differ by Tukey test (α = 0.05). SEM: standard error of the mean.

**Table 3:** Alkaline phosphatase (ALP), total bilirubin (TBIL), aspartate aminotransferase (AST), alanine aminotransferase (ALT), gamma glutamyl transpeptidase (GGT), cholesterol, high-density lipoprotein (HDL), low-density lipoprotein (LDL), very low-density lipoprotein (VLDL), and triglyceride concentrations on blood serum of broiler chickens at 42 days old fed different levels of hydroxytyrosol.

	0 mg HT/kg	5 mg HT/kg	10 mg HT/kg	50 mg HT/kg	*p*-Value	SEM
ALP, U/L	1148.1	955.26	903.75	808.63	0.097	77.68
TBIL, mg/dL	0.057 ^a^	0.046 ^b^	0.035 ^b^	0.037 ^b^	0.0002	0.005
AST, U/L	713.5 ^a^	618.9 ^ab^	639.3 ^a^	451.6 ^b^	0.003	28.32
ALT, U/L	55.90 ^a^	47.92 ^a^	45.75 ^a^	34.33 ^b^	<0.0001	1.867
GGT, U/L	21.30 ^a^	20.58 ^a^	19.00 ^a^	12.67 ^b^	<0.0001	0.833
Cholesterol, mg/dL	125.11	130.83	132.25	126.00	0.496	1.947
HDL, mg/dL	87.20	93.54	91.83	90.82	0.369	1.294
LDL, mg/dL	29.92	26.60	28.22	26.20	0.673	1.162
VLDL, mg/dL	11.77	12.63	12.20	11.38	0.189	0.214
Triglyceride, mg/dL	58.78	63.18	61.00	56.92	0.185	1.070

^a,b^ Means followed by different letters in the same row differ by Tukey test (α = 0.05). SEM: standard error of the mean.

**Table 4:** Lipid content (expressed as g/100 g in wet meat) and fatty acid profile (expressed as g/100 g), peroxide index (meq/kg) and malondialdehyde (mg/kg of wet meat) of breast meat from broiler chickens fed different levels of hydroxytyrosol at 42 days old.

	0 mg HT/kg	5 mg HT/kg	10 mg HT/kg	50 mg HT/kg	*p*-Value	SEM
Lipid content	1.929 ^a^	1.565 ^ab^	1.303 ^ab^	0.933 ^b^	0.022	0.121
Palmitic acid (C16:0)	0.576 ^a^	0.458 ^ab^	0.366 ^ab^	0.294 ^b^	0.015	0.032
Stearic acid (C18:0)	0.180 ^a^	0.167 ^ab^	0.128 ^ab^	0.116 ^b^	0.025	0.008
Oleic acid (C18:1n9c)	0.820 ^a^	0.580 ^ab^	0.488 ^ab^	0.336 ^b^	0.004	0.049
Linoleic acid (C18:2n6c)	0.372 ^a^	0.108 ^b^	0.162 ^b^	0.090 ^b^	<0.0001	0.032
Saturated fatty acid (SFA) ^1^	0.796 ^a^	0.637 ^ab^	0.500 ^ab^	0.438 ^b^	0.024	0.044
Monounsaturated fatty acid (MUFA)	0.893 ^a^	0.618 ^ab^	0.519 ^b^	0.352 ^b^	0.003	0.054
Polyunsaturated fatty acid (PUFA)	0.411 ^a^	0.113 ^b^	0.168 ^b^	0.090 ^b^	<0.0001	0.035
Cis-monounsaturated fatty acid (cis-MUFA)	0.915 ^a^	0.718 ^ab^	0.626 ^b^	0.495 ^b^	0.003	0.046
Omega 6 fatty acids (ω-6)	0.369 ^a^	0.108 ^b^	0.162 ^b^	0.092 ^b^	<0.0001	0.031
Omega 9 fatty acids (ω-9)	0.827 ^a^	0.582 ^ab^	0.488 ^ab^	0.336 ^b^	0.004	0.049
Total fatty acids ^3^	2.030 ^a^	1.481 ^ab^	1.230 ^ab^	0.880 ^b^	0.009	0.114
Unsaturated fatty acid ^2^	1.192 ^a^	0.829 ^ab^	0.705 ^ab^	0.390 ^b^	0.004	0.079
Peroxide index	3.846	4.580	2.923	5.374	0.323	0.468
Malondialdehyde	5.288 ^a^	4.317 ^ab^	5.412 ^a^	3.630 ^b^	0.018	0.236

^a,b^ Means followed by different letters in the same row differ by Tukey test (α = 0.05). SEM: standard error of the mean. ^1^ SFA = sum of C4:0 + C6:0 + C8:0 + C10:0 + C11:0 + C12:0 + C13:0 + C14:0 + C15:0 + C16:0 + C17:0 + C18:0 + C20:0 + C21:0 + C22:0 + C23:0 + C24:0. ^2^ Unsaturated fatty acid = sum of C15:1n5 + C16:1 + C17:1n9 + C18:1n9t + C18:1n9c + C18:2n6c + C18:3n6 + C20:1n9 + C18:3n3 + C20:2n6 + C20:3n6 + C22:1n9 + C20:3n3 + C20:4n6 + C22:2 + C20:5n3 + C24:1n9 + C22:6n3. ^3^ Total fatty acids = sum of saturated fatty acids + unsaturated fatty acids. The cited FA and not presented in Table 4 can be found in Supplementary Materials (Table S1).

## Data Availability

Data are contained within the article and Supplementary Materials.

## Supplementary Materials

The following supporting information can be downloaded at: https://www.mdpi.com/article/10.3390/ani14010119/s1, Table S1: Fatty acids (expressed as g/100 g) of breast meat from broiler chickens fed different levels of hydroxytyrosol at 42 days old.
