# Supplementary material for: Effects of Hydroxytyrosol Supplementation on Performance, Fat and Blood Parameters of Broiler Chickens
Source: Animals (Basel). 2023 Dec 28;14(1):119. doi: 10.3390/ani14010119 (PMC10778069; doi:10.3390/ani14010119)
Supplement: Supplementary file 1 [file animals-14-00119-s001.zip › animals-2788532-supplementary.pdf]

Table S1. Fatty acids (expressed as g/100g) of breast meat from broiler chickens fed different levels of hydroxytyrosol at 42 days old.

	**0 mg HT/ kg**	**5 mg HT/kg**	**10 mg HT/ kg**	**50 mg HT/ kg**	***p-Value***
Butyric acid (C4:0)	<0.10	<0.10	<0.10	<0.10	0.40
Caproic acid (C6:0)	<0.10	<0.10	<0.10	<0.10	0.40
Caprylic acid (C8:0)	<0.10	<0.10	<0.10	<0.10	0.40
Capric acid (C10:0)	<0.10	<0.10	<0.10	<0.10	0.40
n-Undecylic acid (C11:0)	<0.10	<0.10	<0.10	<0.10	0.40
Lauric acid (C12:0)	<0.10	<0.10	<0.10	<0.10	0.40
n-Tridecylic acid (C13:0)	<0.10	<0.10	<0.10	<0.10	0.40
Myristic acid (C14:0)	<0.10	<0.10	<0.10	<0.10	0.40
Myristoleic acid (C14:1)	<0.10	<0.10	<0.10	<0.10	0.40
Pentadecylic acid (C15:0)	<0.10	<0.10	<0.10	<0.10	0.40
Cis-10-Pentadecenoic acid (C15:1n5)	<0.10	<0.10	<0.10	<0.10	0.40
Palmitoleic acid (16:1)	<0.10	<0.10	<0.10	<0.10	0.40
Margaric acid (C17:0)	<0.10	<0.10	<0.10	<0.10	0.40
Cis-8-Heptadecenoic (C17:1n9)	<0.10	<0.10	<0.10	<0.10	0.40
Elaidic acid (C18:1n9t)	<0.10	<0.10	<0.10	<0.10	0.40
Linolelaidic acid (C18:2n6t)	<0.10	<0.10	<0.10	<0.10	0.40
Arachidic acid (C20:0)	<0.10	<0.10	<0.10	<0.10	0.40
gamma-Linolenic acid (C18:3n6)	<0.10	<0.10	<0.10	<0.10	0.40
Cis-11-Eiconosenoic acid (C20:1n9)	<0.10	<0.10	<0.10	<0.10	0.40
alfa-Linolenic acid (C18:3n3)	<0.10	<0.10	<0.10	<0.10	0.40
n-Heneicosylic acid (C21:0)	<0.10	<0.10	<0.10	<0.10	0.40
Cis-11,14-Eicosenoic acid (C20:2n6)	<0.10	<0.10	<0.10	<0.10	0.40
Behenic acid (C22:0)	<0.10	<0.10	<0.10	<0.10	0.40
Cis-11,14,17-Eicosatrienic (C20:3n6)	<0.10	<0.10	<0.10	<0.10	0.40
Erucic acid (C22:1n9)	<0.10	<0.10	<0.10	<0.10	0.40
Dihomo-gamma-Linolenic acid (C20:3n3)	<0.10	<0.10	<0.10	<0.10	0.40
Arachidonic acid (C20:4n6)	<0.10	<0.10	<0.10	<0.10	0.40
Tricosylic acid (C23:0)	<0.10	<0.10	<0.10	<0.10	0.40

Docosadienoic acid (C22:2)	<0.10	<0.10	<0.10	<0.10	0.40
Lignoceric acid (24:0)	<0.10	<0.10	<0.10	<0.10	0.40
Cis-5,8,11,14,17-Eicosapentaenoic acid (EPA: C20:5n3)	<0.10	<0.10	<0.10	<0.10	0.40
Nervonic acid (C24:1n9)	<0.10	<0.10	<0.10	<0.10	0.40
Cis-4,7,10,13,16,19-Docosahexaenoic acid (DHA: C22:6n3)	<0.10	<0.10	<0.10	<0.10	0.40
Trans monounsaturated fatty acid	<0.50	<0.50	<0.50	<0.50	0.40
Trans polyunsaturated fatty acid	<0.50	<0.50	<0.50	<0.50	0.40
Trans fatty acids	<0.10	<0.10	<0.10	<0.10	0.40
Omega 3 fatty acids	<0.10	<0.10	<0.10	<0.10	0.40
